# Multi-horizon forecasting for decision support in a thermally delayed biomass-fired boiler system

**DOI:** 10.1371/journal.pone.0361121

**Published:** 2026-10-23

**Authors:** Sunyong Park, Jiwook Yang, Sungyeol Kim, Kyojung Hwang, JaeJung Lee, Sunhwa Ryu

**Affiliations:** 1 Forest Industrial Materials Division, National Institute of Forest Science, Dongdaemungu, Seoul, Republic of Korea; 2 Department of Biosystems Engineering, Kangwon National University, Chuncheon, Republic of Korea; NED University of Engineering and Technology, PAKISTAN

## Abstract

Thermally delayed biomass-boiler systems exhibit heterogeneous temporal responses that complicate forecasting and operational warning. This study analysed long-term field data from a biomass-fired boiler system to characterise thermal-response lags and evaluate multi-horizon forecasting of the P1 thermal-storage-tank temperature at 60-, 120-, and 180-min horizons. Robust cross-correlation analysis showed statistically detectable but weak associations between P1 temperature and the combustion-related operational inputs, while daily lag estimates exhibited broad and heterogeneous distributions, indicating that the system could not be represented by a single deterministic response time. Benchmark comparison showed that increasing model complexity did not necessarily improve forecasting performance. The Linear AR model achieved the lowest test RMSE and highest R^2^ at all three horizons, with RMSE values of 1.3774, 2.1605, and 2.6182 °C and R^2^ values of 0.7809, 0.4616, and 0.2104 at 60, 120, and 180 min, respectively. Lag coverage increased with forecasting horizon, whereas predictive accuracy progressively decreased, demonstrating a trade-off between pointwise accuracy and coverage of delayed thermal responses rather than a universally optimal horizon. Retrospective out-of-band warning analysis further showed that XGBoost recall decreased from 0.5343 at 60 min to 0.1761 at 120 min and 0.0538 at 180 min, while specificity increased from 0.9095 to 0.9546 and 0.9854, respectively. These results indicate that longer-horizon forecasts became increasingly conservative and detected progressively fewer future out-of-band states. Overall, the findings demonstrate that simple temporal models remain highly competitive for this strongly persistent thermal system and that multi-horizon forecasts should be interpreted as predictive risk indicators rather than evidence of causal improvements in boiler control or operational performance.

## Introduction

Industrial thermal systems often operate under delayed and heterogeneous temperature responses caused by coupled combustion, heat transfer, circulation, and thermal storage processes [1]. These distributed delays complicate stable operation because control actions and measurable temperature responses are not aligned on a single time scale. In practice, this makes it difficult to determine how far ahead forecasts remain sufficiently reliable to provide useful information on future thermal-state risk.

From a classical process-control perspective, boiler systems rarely follow a single dominant time constant. Instead, their dynamic responses are shaped by multiple interacting physical processes, including combustion development, gas-flow propagation, heat transfer through internal components, and heat accumulation within the boiler structure and circulating medium [2]. Dynamic studies of boiler systems have shown that thermal response under load variation is strongly influenced by coupled flow and heat-transfer processes, while biomass boilers additionally exhibit delayed behaviour associated with heat storage and transient exchange within the boiler body and working fluid [3–6]. These characteristics indicate that biomass boiler operation should be understood as a multi-timescale thermal process shaped by distributed delay and structural heat storage rather than by a single fixed response constant.

In recent years, machine-learning techniques have been increasingly applied to boiler prediction and combustion-related optimisation [7]. Previous studies have shown that data-driven models can successfully predict industrial-boiler variables such as exhaust-gas temperature and emissions from real operational data [8–10]. More broadly, forecasting studies in thermal-energy systems have demonstrated that data-driven models can capture complex operating patterns, but their value depends not only on predictive accuracy but also on whether the resulting forecasts can support practical operation and planning [11]. Nevertheless, most previous studies have focused primarily on minimising statistical prediction errors, such as root mean square error and mean absolute error, whereas operationally relevant performance indicators, including temperature-band adherence and out-of-band duration, have rarely been evaluated alongside predictive models.

Meanwhile, multi-step and multi-horizon forecasting have been widely studied in the time-series literature. Existing work has shown that forecasting performance is inherently horizon-dependent and that different strategies may behave differently as the forecast distance increases, especially under complex nonlinear dynamics [12–15]. This issue is particularly important for thermally delayed industrial systems, in which forecast usefulness depends not only on pointwise accuracy but also on predictive lead time and operational actionability. Accordingly, a multi-horizon framework is relevant to biomass boiler monitoring because short-horizon forecasts may provide more accurate estimates of near-term thermal states, whereas longer horizons may provide earlier but potentially less reliable warnings of future thermal deviation [16,17].

To address these issues, this study proposes a thermal-inertia-aware, multi-horizon forecasting framework for operational decision support in a biomass-fired boiler system. Using long-term field operation data, lagged associations between combustion-related inputs and the P1 thermal-storage-tank temperature were first characterised using robust time-series analysis. Multi-horizon temperature forecasts were then developed for 60-, 120-, and 180-min horizons and benchmarked against simple statistical and machine-learning models. The practical relevance of these forecasts was further examined through retrospective detection of future out-of-band temperature states. Accordingly, this study aimed to (i) characterise the distributed temporal response of the biomass boiler system, (ii) quantify horizon-dependent forecasting performance relative to appropriate baseline models, and (iii) evaluate the usefulness and limitations of multi-horizon forecasts as retrospective warning indicators. The framework was evaluated as an offline decision-support approach and was not interpreted as evidence of causal control intervention or autonomous boiler operation.

## Materials and methods

### Boiler system description

The study was conducted at the Damba-u Energy Centre in Goesan-gun, Republic of Korea, using an industrial biomass-fired hot-water boiler (M400, HDG Bavaria, Germany) with a nominal thermal capacity of 400 kWth. Heat generated from biomass combustion was transferred to circulating water through the boiler heat-exchange system and subsequently stored in a 40 m^3^ thermal storage tank before being supplied to the downstream heating network. The approximate total water volume of the boiler, thermal storage tank, and circulation network was 70 m^3^. Owing to this large water inventory and thermal-storage capacity, changes in combustion conditions are not immediately reflected in the storage-tank temperature, resulting in delayed and buffered thermal responses. A schematic representation of the boiler, circulation loop, thermal storage tank, and associated energy-supply system is shown in Fig 1.

**Fig 1:**
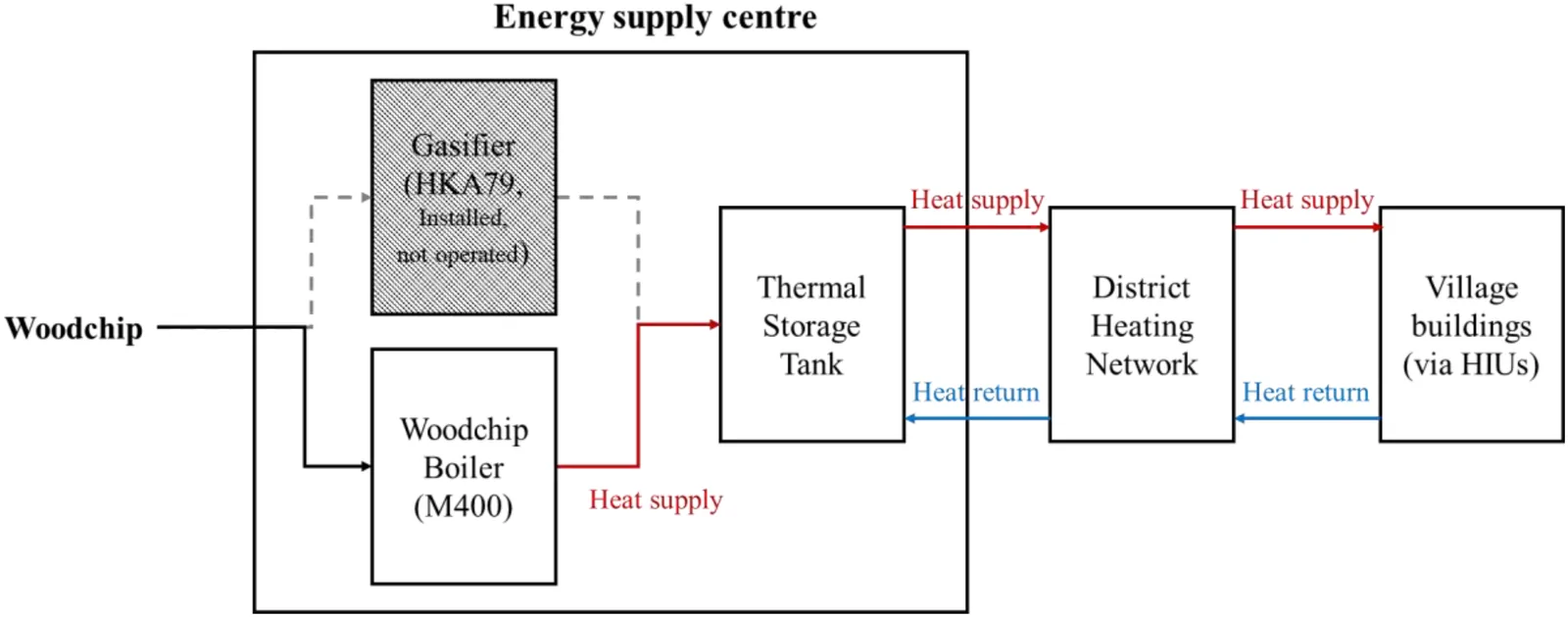
Schematic diagram of the forest-biomass energy-autonomous village CHP facility (Damba-u Energy Centre, Goesan-gun, Republic of Korea).

The P1 temperature sensor installed in the thermal storage tank was selected as the primary variable representing the thermal state of the system. Because P1 represents the temperature of accumulated hot water rather than the instantaneous combustion-zone temperature, its response reflects the combined effects of biomass combustion, heat transfer through the boiler, water circulation, thermal storage, and downstream heat demand. Detailed circulation-flow measurements and manufacturer-specified P1 sensor accuracy were not available in the archived operational dataset and were therefore not explicitly incorporated into the present analysis.

Combustion operation was characterised using the biomass fuel-feed setting and the primary- and secondary-air blower settings, denoted as Air1 and Air2, respectively. These variables were recorded by the boiler control system as relative actuator commands expressed in percentage units. Accordingly, the fuel variable was treated as a fuel-feed setting rather than an absolute biomass mass-flow rate, and Air1 and Air2 were treated as blower settings rather than directly measured volumetric airflow rates. No validated site-specific conversion from the fuel-feed percentage to biomass mass flow was available. The principal variables used in the lag analysis and forecasting models are summarised in Table 1.

**Table 1:** Process variables used in the lag analysis and forecasting models.

Variable	Description	Unit	Role
**Fuel-feed setting**	Relative biomass fuel-feed command recorded by the boiler controller	%	Combustion heat-input setting
**Primary-air blower setting (Air1)**	Relative primary-air blower command used for combustion control	%	Primary combustion-air control
**Secondary-air blower setting (Air2)**	Relative secondary-air blower command used for combustion control and mixing	%	Secondary combustion-air control
**P1 temperature**	Temperature of the thermal storage tank representing the accumulated hot-water thermal state	°C	Target thermal-state variable
**External temperature**	Ambient temperature around the boiler facility	°C	Environmental/contextual variable

Direct measurements of downstream heat demand and circulation flow were not available in the archived dataset. These unobserved variables may affect both combustion-input settings and P1 temperature and are therefore recognised as potential confounding factors when interpreting the observed lag relationships. External ambient temperature was retained as an available contextual variable representing environmental influences on the thermal system.

### Data acquisition and preprocessing

Operational data were obtained from the boiler monitoring and control system at the Damba-u Energy Centre from 4 September 2024–31 March 2025. Seven monthly data files were combined, yielding 1,781,450 raw records before preprocessing. Records outside the predefined analysis period were removed, and the measurements were temporally aligned and aggregated to a uniform 1-min resolution using the mean of all available observations within each minute. This procedure produced a continuous analysis timeline of 300,960 min covering 209 calendar days.

The principal variables retained for analysis were P1, Fuel, Air1, Air2, and external temperature. Basic quality-control ranges of 0–100 °C for P1, −40–60 °C for external temperature, and 0–100% for Fuel, Air1, and Air2 were applied. No observations of these variables fell outside the specified ranges, and no duplicate or invalid timestamps remained after data integration.

Missing observations were retained as missing values rather than interpolated to avoid introducing artificial temporal information. After 1-min aggregation, P1 was unavailable for 7,685 min, corresponding to 2.55% of the complete analysis timeline. Valid P1 measurements were available on 206 of the 209 calendar days. Rows with unavailable forecast targets were excluded from the corresponding modelling dataset. A forecast-issuance row was retained only when no more than 30% of the candidate predictor features were missing. No forward-looking interpolation or imputation using future observations was performed.

Startup, shutdown, and low-activity periods were not automatically excluded from the primary forecasting dataset solely on the basis of operating status. Instead, an auxiliary operating-state label was generated from the available combustion variables for sensitivity assessment. Thus, the forecasting dataset retained the range of operating conditions contained in the archived field data rather than being restricted to manually selected steady-state periods.

For model development and evaluation, the dataset was divided strictly in chronological order into training, validation, and independent test subsets using an approximate 70:15:15 allocation. To minimise temporal leakage associated with the strong autocorrelation of the 1-min time series and overlapping forecast targets, a 180-min embargo, corresponding to the maximum forecasting horizon, was inserted between adjacent subsets. The training period ended on 30 January 2025 at 02:18, the validation period began on 30 January 2025 at 05:18 and ended on 1 March 2025 at 13:25, and the test period began on 1 March 2025 at 16:25. Forecast targets were also required to remain entirely within the temporal boundary of their respective subset.

Because the number of valid forecast targets differed slightly with forecasting horizon, the final sample sizes also varied. For the 60-min horizon, the training, validation, and test sets contained 204,027, 43,624, and 43,595 samples, respectively. The corresponding sample sizes were 203,727, 43,624, and 43,535 for the 120-min horizon and 203,484, 43,624, and 43,475 for the 180-min horizon. All subsequent feature construction, model training, model selection, and performance evaluation were conducted using these temporally separated datasets.

### Robust thermal lag analysis

To characterise the delayed temporal relationships between combustion-related inputs and the P1 thermal-storage-tank temperature, a robust lag-analysis procedure was applied. Prior to cross-correlation analysis, the P1 temperature and each operational input series were first differenced to reduce the influence of long-term trends and level shifts. The first-differenced signals were subsequently smoothed using a 5-min moving window. Stationarity of the processed time series was assessed using both the augmented Dickey–Fuller (ADF) test and the Kwiatkowski–Phillips–Schmidt–Shin (KPSS) test.

For a variable *x*_*t*_, the first-differenced series was calculated as:


$$\begin{array}{c} \Delta x_{t}=x_{t}-x_{t-\text{1}} \end{array}$$ (1)

where *x*_*t*_ is the observed value at time *t*, and *Δx*_*t*_ represents the change from the preceding time step.

Cross-correlation analysis was then performed between the processed P1 temperature series and each of the three combustion-related operational variables: fuel-feed setting, primary-air blower setting (Air1), and secondary-air blower setting (Air2). Positive lag values were defined such that a change in the operational input preceded the corresponding change in P1 temperature. Lag values from 10 to 360 min were evaluated. Lags shorter than 10 min were excluded to reduce the influence of near-synchronous controller actions, sensor synchronisation effects, and short-term measurement noise.

For each input variable *X*, the lagged Pearson correlation with the P1 temperature series *T* was calculated as:


$$\begin{array}{c} r_{XY}(\tau )=corr[\Delta X(t),\Delta T(t+\tau )] \end{array}$$ (2)

where *τ* denotes the positive time lag in minutes, *ΔX(t)* is the processed change in the operational input, and *ΔT(t + τ)* is the corresponding processed change in P1 temperature at lag τ.

For descriptive lag screening, the response-associated lag was identified as the lag showing the largest absolute cross-correlation coefficient within the predefined lag range:


$$\begin{array}{c} \tau_{assoc}=\text{arg}\underset{\text{10}\leq \tau \leq \text{360}}{\text{max}}\left|r_{XY}(\tau )\right| \end{array}$$ (3)

The sign of the corresponding correlation coefficient was retained separately to distinguish positive and negative temporal associations. The resulting lag was interpreted as a statistical response-associated lag rather than as a deterministic physical time constant.

Because serially correlated time-series data can produce apparently large cross-correlation coefficients even in the absence of a robust lagged relationship, statistical significance was assessed using a circular-shift max-statistic permutation test. For each operational variable, the input series was circularly shifted while preserving its internal serial structure, and the maximum absolute cross-correlation across the entire 10–360 min lag range was recalculated. This procedure was repeated 200 times to generate a null distribution of the maximum correlation statistic. The observed maximum absolute correlation was then compared with this null distribution.

In addition to the full-period analysis, lag estimation was performed separately for individual operating days to quantify day-to-day heterogeneity in thermal response. Daily analyses were retained only when at least 480 valid paired observations were available and when sufficient variation was present in the corresponding operational input. For each retained day, the response-associated lag and its corresponding correlation coefficient were recorded. The resulting daily lag distributions were subsequently compared with the 60-, 120-, and 180-min forecasting horizons.

### Multi-horizon forecasting models and benchmark evaluation

The forecasting task was defined as the direct prediction of the future P1 thermal-storage-tank temperature at three forecasting horizons: 60, 120, and 180 min. Separate models were developed independently for each horizon so that the temperature at time *t + h* was predicted using only information available at the forecast-issuance time \(t\) or earlier. The forecasting problem was expressed as:


$$\begin{array}{c} {\hat{T}}_{P\text{1}}(t+h)=f_{h}(X_{t}text\;h\in \{\text{6}\text{0},\text{1}\text{2}\text{0},\text{1}\text{8}\text{0}|min\} \end{array}$$ (4)

where, ${\hat{T}}_{P\text{1}}(t+h)$ represents the predicted temperature at the prediction horizon *h*, and *X*_*t*_ denotes the vector of input variables including fuel feed rate, primary air setting, secondary air setting, and contextual variables.

Five forecasting approaches were evaluated at each horizon: a historical-mean baseline, a persistence baseline, a linear autoregressive (Linear AR) model, a random-forest (RF) regression model, and an XGBoost regression model. The historical-mean baseline predicted the future P1 temperature using the mean P1 temperature of the model-development data. The persistence baseline assumed that the future P1 temperature remained equal to the current temperature observed at forecast issuance, i.e., ${\hat{T}}_{P\text{1}}(t+h)=T_{P\text{1}}(t)$. The Linear AR model represented a simple time-series benchmark based on the current and lagged P1 temperatures. RF and XGBoost were included as nonlinear machine-learning approaches to evaluate whether multivariable nonlinear modelling provided additional predictive value beyond simple thermal persistence and autoregressive structure.

The multivariable predictor set consisted of five measured variables: P1 temperature, fuel-feed setting, primary-air blower setting (Air1), secondary-air blower setting (Air2), and ambient temperature. For each variable, the value at forecast issuance time *t* and lagged values at 5-min intervals from 5 to 180 min were generated. Thus, each variable contributed 37 predictors (the current value plus 36 lagged values), resulting in a total of 185 candidate predictors. All lagged predictors were constructed exclusively from observations recorded at or before forecast issuance time *t*; no observations occurring after the prediction time were included in the feature matrix.

For each process variable *z*, the lagged predictor vector was defined as:


$$\begin{array}{c} z_{t}=[z_{t},\;z_{t-\text{5}},z_{t-\text{1}\text{0}},\ldots ,z_{t-\text{1}\text{8}\text{0}}] \end{array}$$ (5)

where z∈{P1, Fuel, Air1, Air2, Ambient}.

For the Linear AR model, the current P1 temperature and its lagged values were used as predictors and ridge regression was applied after median imputation and standardisation. The RF and XGBoost models used the complete multivariable predictor set. Missing predictor values remaining after temporal alignment were handled within the model-development pipeline; median imputation was applied for the Linear AR and RF models, whereas XGBoost handled missing predictor values internally. To avoid training models on observations with excessive missing information, only forecast-issuance records for which the current P1 value and future target were available and the missing fraction of the predictor set did not exceed 30% were retained.

XGBoost hyperparameters were selected using the validation set, with the configuration yielding the lowest validation RMSE retained for each forecasting horizon. The final selected configuration comprised 1,000 trees, a learning rate of 0.02, a maximum tree depth of 4, subsample and column-sampling ratios of 0.85, an L1 regularisation coefficient of 0.1, an L2 regularisation coefficient of 5.0, a minimum child weight of 5, and gamma of 0. The squared-error regression objective was used with a random seed of 45. After hyperparameter selection, the final model was refitted using the combined training and validation data and evaluated once on the independent test set. The RF model was fitted using 300 regression trees, a minimum leaf size of 2, square-root feature subsampling, and a random seed of 45. The Linear AR model used ridge regression with a regularisation parameter of α = 1.0.

Forecasting performance was evaluated using the root-mean-square error (RMSE), mean absolute error (MAE), and coefficient of determination (R^2^). These metrics were calculated as follows:


$$\begin{array}{c} RMSE=\sqrt{\frac{\text{1}}{n}\sum_{i=\text{1}}^{n}{\left(y_{i}-\hat{y_{i}}\right)}^{\text{2}}} \end{array}$$ (7)

$$\begin{array}{c} MAE=\frac{\text{1}}{n}\sum_{i=\text{1}}^{n}\left|y_{i}-\hat{y_{i}}\right| \end{array}$$ (8)

$$\begin{array}{c} R^{\text{2}}=\text{1}-(\frac{\sum_{i=\text{1}}^{n}{\left(y_{i}-\hat{y_{i}}\right)}^{\text{2}}}{\sum_{i=\text{1}}^{n}{\left(y_{i}-\hat{y_{i}}\right)}^{\text{2}}} \end{array}$$ (9)

where *y*_*i*_ is the observed P1 temperature, $\hat{{\text{y}}_{\text{i}}}$ is the corresponding predicted temperature, $\overset{―}{y}$ is the mean of the observed temperatures, and *n* is the number of evaluated observations. Lower RMSE and MAE values indicate smaller prediction errors, whereas an R^2^ value closer to 1 indicates greater explanatory performance relative to the observed variation.

Model and hyperparameter selection were based exclusively on the validation set. The independent test set was not used for model selection, hyperparameter tuning, feature construction, or definition of the temporal partitions. Test-set performance was reported only after the model-development procedure had been completed. All models were evaluated on the same horizon-specific test observations to ensure direct comparability among forecasting approaches.

### Retrospective forecast-based warning evaluation

For retrospective evaluation of forecast-based warning performance, an empirical reference band was defined for the P1 thermal-storage-tank temperature [18–20]. To avoid information leakage, the reference band was derived exclusively from the training data and was not recalculated using the validation or test sets. The lower and upper limits were defined as the training-set mean minus and plus one standard deviation, respectively.

Using the revised training dataset, the mean P1 temperature was 62.496 °C and the standard deviation was 3.556 °C, resulting in an empirical reference band of 58.940–66.052 °C. This band was used as a data-derived reference for retrospective warning evaluation and should not be interpreted as an equipment safety limit or manufacturer-specified operating range.

For each forecasting horizon, a predicted out-of-band event was defined when the forecasted P1 temperature fell below the lower reference limit or exceeded the upper reference limit. For descriptive multi-horizon warning analysis, a hierarchical interpretation rule was applied. If the 60-min forecast was outside the reference band, the case was classified as a near-term alert. If the 60-min forecast remained in band but the 120-min forecast was outside the band, the case was classified as an intermediate warning. If both the 60- and 120-min forecasts remained in band but the 180-min forecast was outside the band, the case was classified as an extended watch. Forecasts remaining within the reference band at all three horizons were classified as normal.

Importantly, these warning classifications were evaluated retrospectively using historical observations. No fuel-feed setting, Air1 setting, Air2 setting, or other boiler actuator was modified as part of this analysis, and no counterfactual P1 temperature trajectory was generated following a hypothetical control intervention. The analysis therefore evaluates predictive warning capability rather than causal control performance.

Warning performance was evaluated by comparing the predicted band status with the observed P1 band status at the corresponding future timestamp. The classifications were summarised as true positives, false positives, true negatives, and false negatives, from which precision, recall (sensitivity), specificity, false-alarm rate, and F1 score were calculated.

## Results & discussion

### Thermal lag characteristics and robustness

The revised lag analysis revealed statistically detectable but weak temporal associations between the combustion-related operational inputs and the P1 thermal-storage-tank temperature. For fuel feed, the largest absolute cross-correlation occurred at a lag of 10 min, with a correlation coefficient of *r* = −0.0742. In addition to this short-lag negative association, the fuel-feed series exhibited a weaker positive local peak at approximately 45 min (*r* ≈ 0.06). The Air1 showed its strongest positive association at a lag of 45 min (*r* = 0.0812), whereas the Air2 showed its strongest positive association at 43 min (*r* = 0.0486). The complete cross-correlation functions for the three operational inputs are shown in Fig 2.

**Fig 2:**
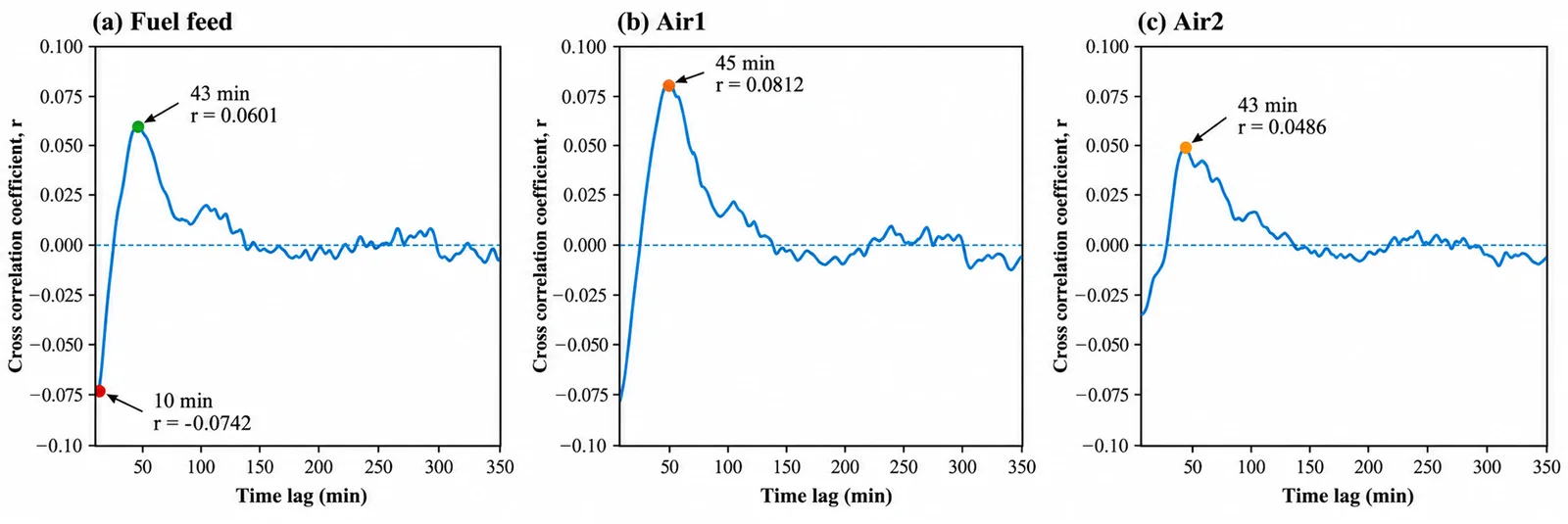
Cross-correlation between P1 temperature and (a) fuel feed, (b) Air1, and (c) Air2 after first differencing and 5-min smoothing.

Despite their small magnitudes, the observed maximum absolute correlations exceeded the corresponding null distributions obtained from the circular-shift max-statistic permutation analysis. The permutation-based *p*-value was 0.005 for fuel feed, Air1, and Air2. These findings indicate that the detected lagged associations were statistically distinguishable from those expected under the permutation null model. However, the relatively small correlation coefficients also demonstrate that the identified lags should not be interpreted as deterministic physical response times or as a single dominant thermal delay. Rather, they represent weak aggregate temporal associations embedded within heterogeneous and feedback-regulated boiler operation.

Stationarity tests further supported the use of the processed time series for the cross-correlation analysis. Following first differencing and 5-min smoothing, the augmented Dickey–Fuller tests rejected the unit-root null hypothesis for P1 temperature, fuel feed, Air1, and Air2 (*p* < 0.001), whereas the KPSS tests did not reject the null hypothesis of stationarity (*p* ≈ 0.10). The consistency of these results indicates that the processed series were suitable for subsequent lag analysis.

Daily lag analysis further demonstrated substantial day-to-day heterogeneity in the temporal response of the system. After applying the predefined data-completeness and input-variation criteria, 126 operating days were retained for fuel feed, 127 days for Air1, and 111 days for Air2. The median response-associated lag was 56 min for fuel feed, 67 min for Air1, and 85 min for Air2, whereas the corresponding mean lag values were 117.8, 124.4, and 133.9 min, respectively. The differences between the median and mean values indicate right-skewed lag distributions, with a subset of operating days exhibiting substantially longer response-associated lags. The daily lag distributions for all three operational inputs are presented in Fig 3.

**Fig 3:**
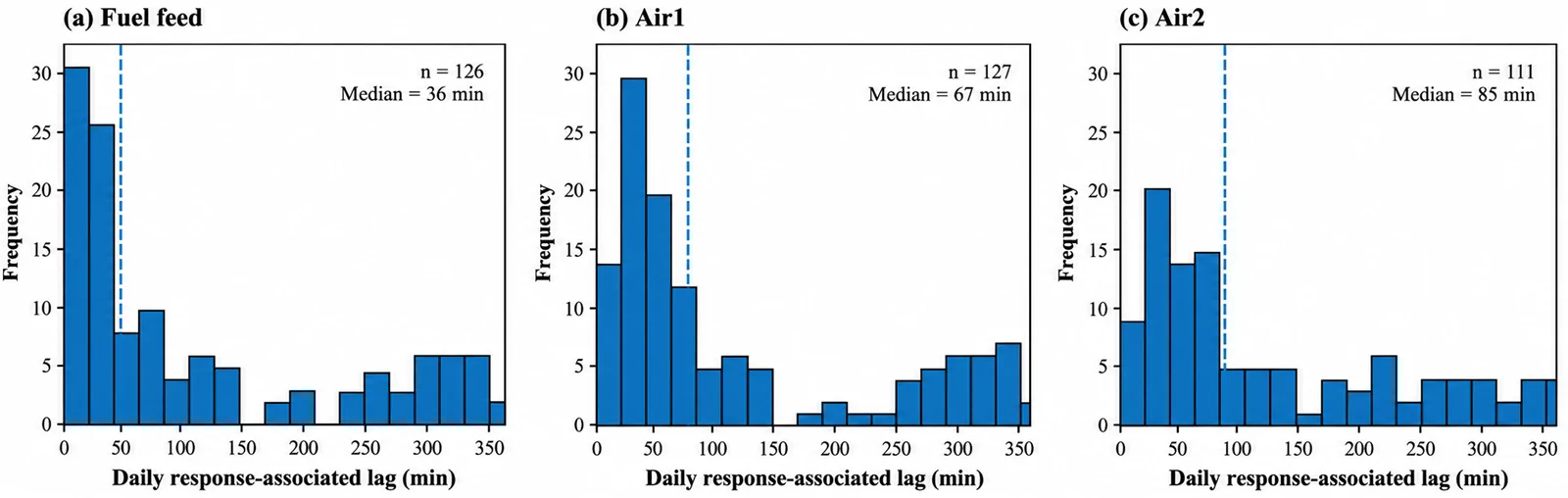
Daily response-associated lag distributions for (a) fuel feed, (b) Air1, and (c) Air2. Dashed lines indicate median lags.

These results demonstrate that the thermal response of the boiler system cannot be represented adequately by a single fixed delay. Instead, the apparent response-associated lag varied considerably among operating days and among fuel-feed and airflow variables. This variability is consistent with the combined effects of biomass feeding, combustion development, airflow regulation, heat transfer, water circulation, thermal storage, and feedback-based boiler operation. Accordingly, the positive peaks observed at approximately 43–45 min in the aggregate analyses should be interpreted as population-level statistical characteristics rather than universal thermal time constants of the system.

When the daily response-associated lag distributions were compared with the forecasting horizons, the 60-min horizon covered 50.8% of retained fuel-feed lags, 46.5% of Air1 lags, and 32.4% of Air2 lags. The corresponding coverage ratios increased to 65.1%, 66.1%, and 60.4% at the 120-min horizon and to 72.2%, 72.4%, and 69.4% at the 180-min horizon, respectively. Thus, increasing the forecasting horizon progressively increased the proportion of observed daily lags covered, although the additional gain in lag coverage became smaller from 120 to 180 min. These results were subsequently considered together with horizon-dependent forecasting performance rather than being used alone to define an optimal forecasting horizon.

### Multi-horizon benchmark forecasting performance

The forecasting performance of the five evaluated models on the independent test dataset is summarised in Table 2. Clear horizon-dependent degradation was observed for all forecasting approaches, with prediction errors increasing as the forecasting horizon increased. However, the benchmark comparison also showed that greater model complexity did not necessarily result in improved predictive performance.

**Table 2:** Test performance of the evaluated forecasting models for P1 temperature.

Horizon (min)	Model	n	RMSE (°C)	MAE (°C)	R²
60	**Linear AR**	43,595	**1.3774**	1.0553	**0.7809**
60	XGBoost	43,595	1.4099	1.1199	0.7704
60	Persistence	43,595	1.4343	**1.0470**	0.7624
60	Random Forest	43,595	1.5280	1.1817	0.7303
60	Historical mean	43,595	3.7745	3.1788	−0.6455
120	**Linear AR**	43,535	**2.1605**	1.6828	**0.4616**
120	Persistence	43,535	2.1721	**1.5620**	0.4558
120	Random Forest	43,535	2.3046	1.7918	0.3874
120	XGBoost	43,535	2.3105	1.8299	0.3843
120	Historical mean	43,535	3.7726	3.1760	−0.6416
180	**Linear AR**	43,475	**2.6182**	2.0312	**0.2104**
180	Persistence	43,475	2.7064	**1.9894**	0.1563
180	XGBoost	43,475	2.7122	2.1155	0.1527
180	Random Forest	43,475	2.7325	2.1094	0.1399
180	Historical mean	43,475	3.7713	3.1739	−0.6382

At the 60-min horizon, the Linear AR model achieved the lowest RMSE (1.3774 °C) and the highest R^2^ (0.7809), followed closely by XGBoost (RMSE = 1.4099 °C, R^2^ = 0.7704) and persistence (RMSE = 1.4343 °C, R^2^ = 0.7624). The persistence model showed the lowest MAE (1.0470 °C), slightly lower than that of Linear AR (1.0553 °C). Thus, although XGBoost provided a small improvement over persistence in terms of RMSE and R^2^, the differences among the three models were relatively small at this short forecasting horizon.

At the 120-min horizon, the Linear AR model again achieved the lowest RMSE (2.1605 °C) and the highest R^2^ (0.4616). Persistence produced a nearly comparable RMSE of 2.1721 °C and the lowest MAE of 1.5620 °C. In contrast, XGBoost yielded an RMSE of 2.3105 °C and an R^2^ of 0.3843, indicating that its nonlinear multivariable structure did not provide additional predictive benefit over the simpler temporal baselines at this horizon.

A similar pattern was observed at the 180-min horizon. Linear AR produced the lowest RMSE (2.6182 °C) and the highest R^2^ (0.2104), while persistence achieved the lowest MAE (1.9894 °C). The corresponding XGBoost performance was RMSE = 2.7122 °C, MAE = 2.1155 °C, and R^2^ = 0.1527. Although positive R^2^ values were retained by the principal forecasting models, the substantial reduction in explanatory performance indicates that reliable pointwise prediction became increasingly difficult as the forecasting horizon approached 180 min.

The comparative RMSE results are illustrated in Fig 4. Across all three forecasting horizons, Linear AR consistently produced the lowest RMSE, whereas the historical-mean baseline showed substantially larger errors. The differences between Linear AR, persistence, and XGBoost were relatively small at 60 min but became more apparent as the forecasting horizon increased.

**Fig 4:**
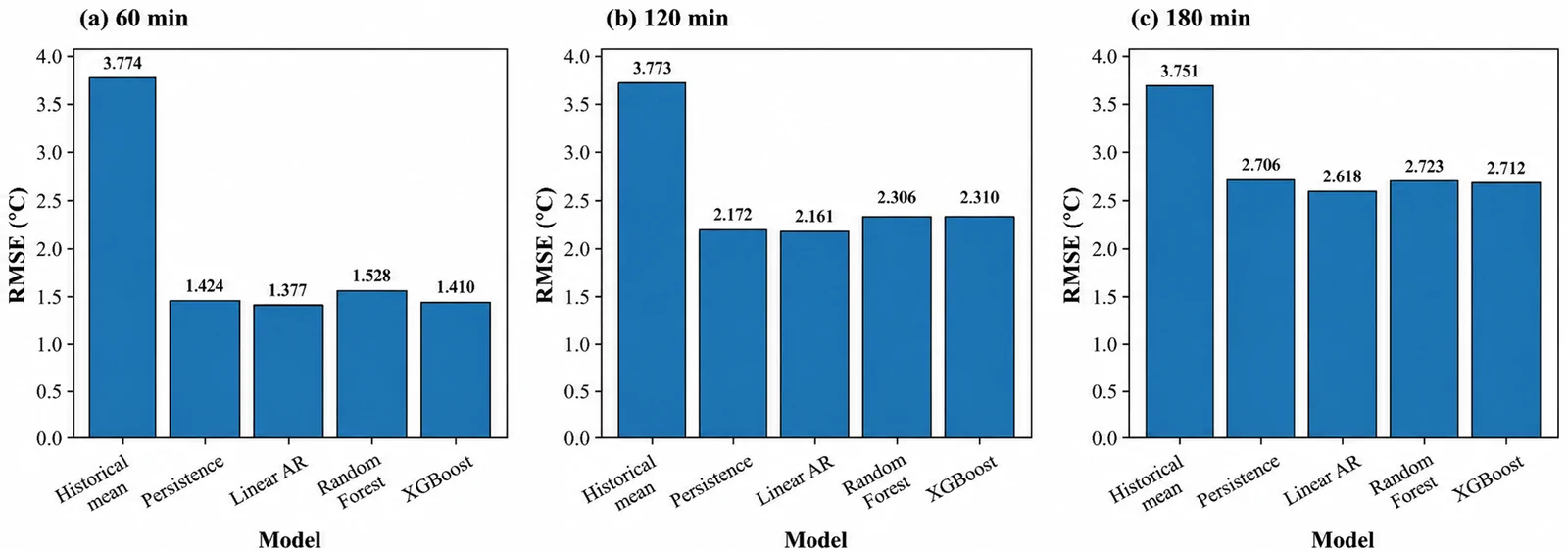
Test RMSE of the forecasting models at (a) 60-, (b) 120-, and (c) 180-min horizons.

Overall, these results demonstrate that the strong thermal persistence of the storage system accounted for a substantial proportion of its forecastability. The consistently competitive performance of the persistence and Linear AR models indicates that recent P1 temperature history contains much of the information required for short- and medium-term prediction. The benefit of the nonlinear XGBoost model was limited primarily to the 60-min horizon and disappeared at longer horizons. Therefore, model complexity alone should not be regarded as evidence of improved forecasting capability in this thermally delayed system.

The historical-mean baseline yielded negative R^2^ values at all three horizons, confirming that the forecasting models extracted temporal information beyond a stationary mean estimate. However, the relatively strong performance of the persistence benchmark highlights the highly autocorrelated nature of the P1 thermal state. This finding also explains why the difference between the more complex machine-learning models and simple temporal baselines was modest, particularly at the longer forecasting horizons.

The horizon-dependent deterioration of the XGBoost forecasts is also evident in the parity plots shown in Fig 5. At 60 min, the predicted and observed temperatures remained relatively closely aligned with the 1:1 line, whereas progressively greater dispersion and systematic deviation were observed at 120 and 180 min. These visual patterns are consistent with the increasing RMSE and decreasing \(R^2\) values obtained as the forecasting horizon increased.

**Fig 5:**
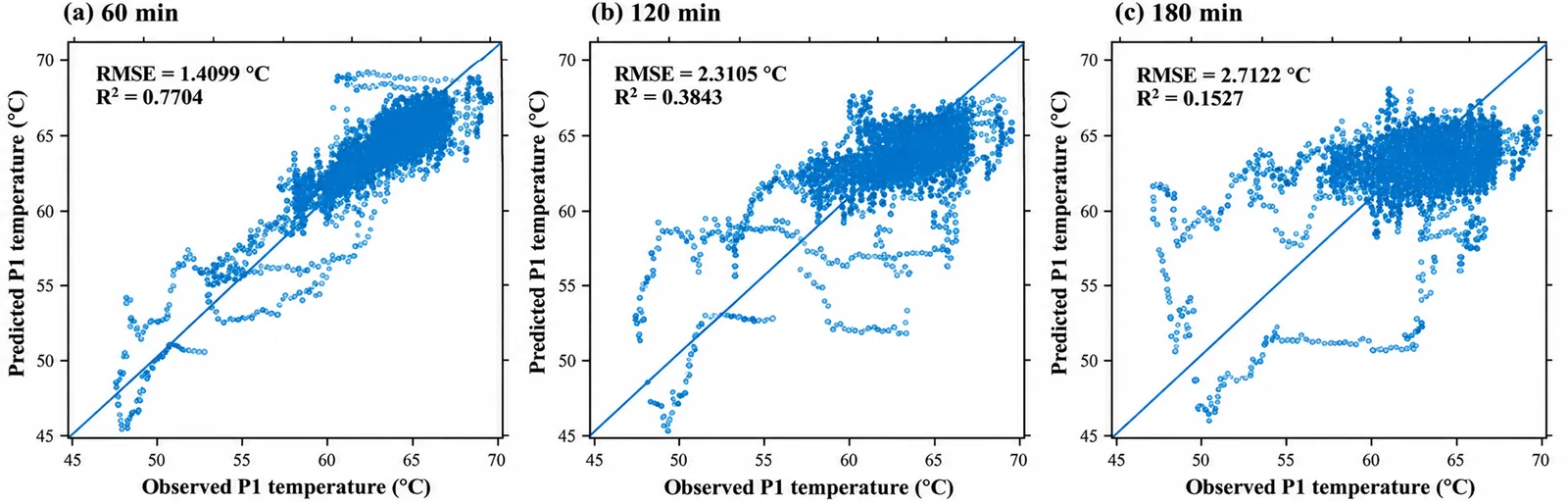
Observed versus predicted P1 temperatures for the XGBoost model at (a) 60-, (b) 120-, and (c) 180-min horizons. The solid line represents the 1:1 reference.

Although XGBoost was not the best-performing model at every forecasting horizon, SHAP analysis was used to examine how the nonlinear multivariable model utilised the available predictors. Across all three horizons, the current P1 temperature was the most influential individual predictor. At the 60-min horizon, P1 had a mean absolute SHAP value of 2.7018, substantially larger than that of the next-ranked feature, P1 lagged by 5 min (0.3564). Similar dominance of the current P1 state was observed at 120 and 180 min, with mean absolute SHAP values of 1.7417 and 1.1780, respectively.

Lagged P1 variables also remained among the most influential predictors, whereas fuel-feed and airflow variables generally showed smaller secondary contributions. At the longer horizons, P1 lagged by 180 min became increasingly influential, ranking third at 120 min and second at 180 min. As shown in Fig 6, the SHAP distributions therefore indicate that the XGBoost forecasts were dominated by the current and historical thermal-storage state, while combustion-related control variables made comparatively smaller contributions. These results reinforce the benchmark-model findings and further demonstrate the importance of temporal persistence in the P1 temperature dynamics. SHAP values were interpreted as measures of predictive contribution and not as evidence of causal effects.

**Fig 6:**
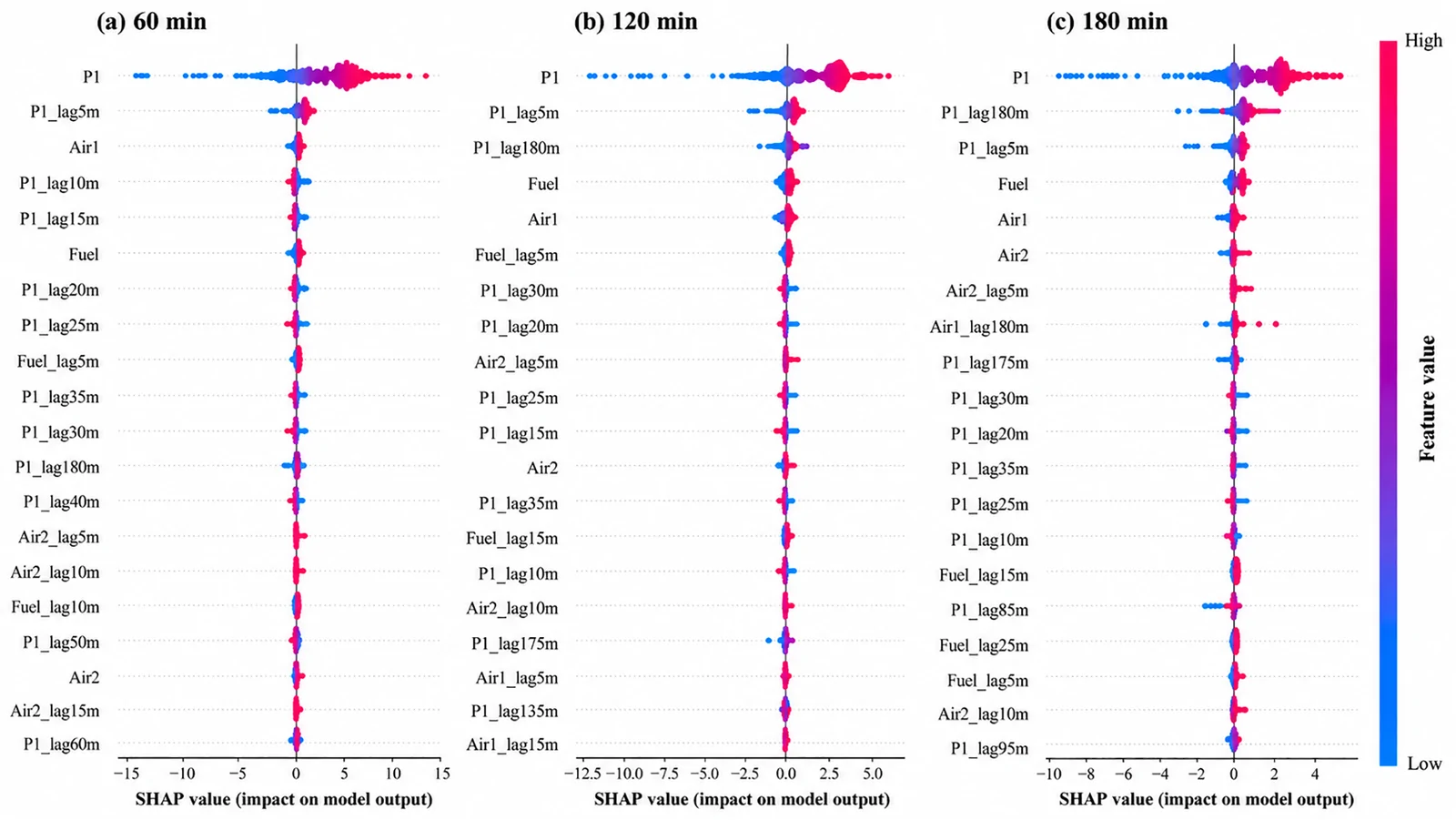
SHAP summary plots for the XGBoost models at (a) 60-, (b) 120-, and (c) 180-min forecasting horizons.

### Prediction horizon adequacy

The adequacy of the 60-, 120-, and 180-min forecasting horizons was evaluated by considering both the coverage of the observed daily response-associated lag distributions and the corresponding forecasting performance. Because the daily lag distributions differed among fuel feed, Air1, and Air2, no single forecasting horizon fully represented the temporal response characteristics of all operational inputs.

At the 60-min horizon, 50.8% of the retained fuel-feed lags, 46.5% of the Air1 lags, and 32.4% of the Air2 lags were covered. Increasing the horizon to 120 min increased the corresponding coverage ratios to 65.1%, 66.1%, and 60.4%, respectively. At 180 min, coverage further increased to 72.2% for fuel feed, 72.4% for Air1, and 69.4% for Air2. Thus, extending the forecasting horizon progressively increased lag coverage, although the additional gain from 120 to 180 min was relatively modest compared with the gain from 60 to 120 min. These horizon-dependent lag coverage ratios are summarised in Fig 7.

**Fig 7:**
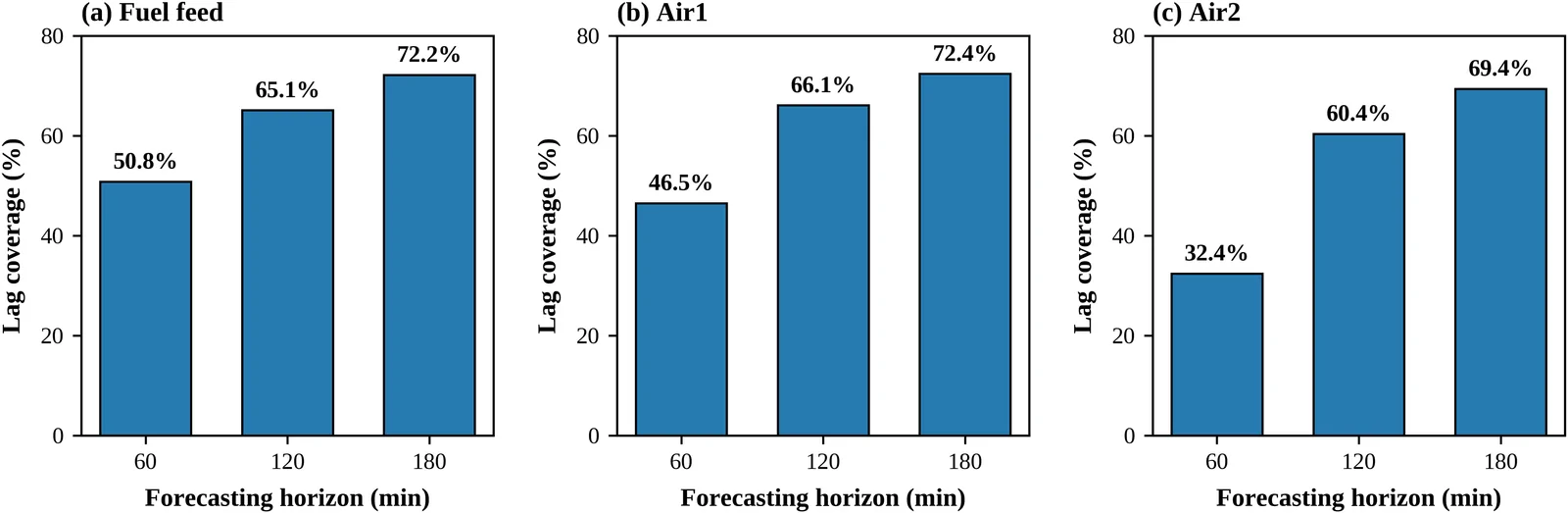
Daily lag coverage at 60-, 120-, and 180-min forecasting horizons for (a) fuel feed, (b) Air1, and (c) Air2.

This improvement in lag coverage was accompanied by a clear deterioration in forecasting accuracy. The best-performing Linear AR model achieved RMSE values of 1.3774, 2.1605, and 2.6182 °C and R^2^ values of 0.7809, 0.4616, and 0.2104 at the 60-, 120-, and 180-min horizons, respectively. Similar horizon-dependent degradation was observed for the other forecasting models. These results demonstrate a clear trade-off between the temporal coverage of delayed thermal responses and pointwise forecasting accuracy.

The 60-min horizon therefore provided the strongest pointwise predictive performance but represented only a limited fraction of the longer daily lag responses, particularly for Air2. The 120-min horizon substantially increased lag coverage while retaining moderate predictive capability, whereas the 180-min horizon provided only a relatively small additional increase in coverage at the cost of a further reduction in prediction accuracy. Accordingly, the 120-min horizon may be regarded as an intermediate forecasting scale when broader lag coverage is required, but it should not be interpreted as universally optimal. The preferred forecasting horizon depends on whether the operational objective prioritises short-term predictive accuracy or broader coverage of delayed system responses.

### Retrospective out-of-band warning performance

The retrospective warning analysis evaluated whether the forecasting models could identify future P1 temperatures outside the empirical reference band without assuming any intervention-induced change in boiler operation. During the independent test period, 95 observed out-of-band episodes were identified, corresponding to a total out-of-band duration of 19,437 min. The median duration of an individual out-of-band episode was 130 min, whereas the longest continuous episode lasted 1,532 min. The horizon-dependent out-of-band warning performance of the XGBoost models is summarised in Fig 8.

**Fig 8:**
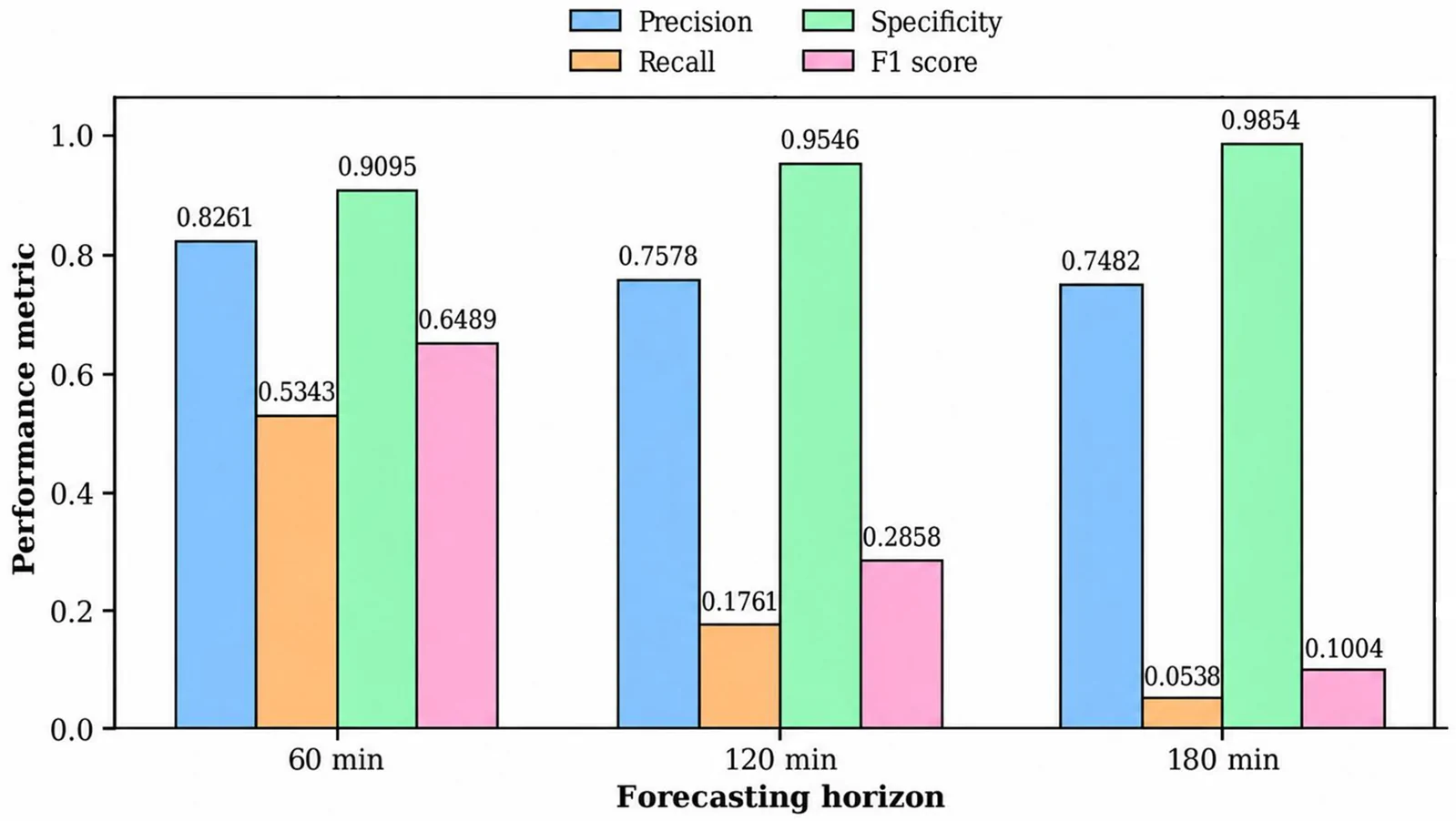
Out-of-band warning performance of the XGBoost models at 60-, 120-, and 180-min forecasting horizons.

For the XGBoost model, out-of-band event-detection performance deteriorated markedly as the forecasting horizon increased. At the 60-min horizon, precision was 0.8261, recall was 0.5343, specificity was 0.9095, the false-alarm rate was 0.0905, and the F1 score was 0.6489. Thus, most warnings issued by the model corresponded to actual future out-of-band states, although approximately half of the observed out-of-band states were not detected.

At the 120-min horizon, XGBoost retained relatively high precision (0.7578) and specificity (0.9546), while the false-alarm rate decreased to 0.0454. However, recall declined sharply to 0.1761, resulting in an F1 score of only 0.2858. This indicates that the apparent reduction in false alarms at the longer horizon was achieved primarily through increasingly conservative warning behaviour rather than improved event discrimination.

This tendency became more pronounced at the 180-min horizon. Precision remained 0.7482 and specificity increased to 0.9854, with a false-alarm rate of only 0.0146, but recall decreased to 0.0538 and the F1 score to 0.1004. Therefore, the 180-min XGBoost forecasts identified only a small fraction of the actual future out-of-band states and should not be interpreted as providing reliable early-warning capability.

The event-detection results also demonstrate that pointwise forecasting accuracy and warning performance should be interpreted separately. Although XGBoost provided competitive pointwise prediction at the 60-min horizon, simpler temporal baselines remained highly competitive for out-of-band event detection. In particular, persistence produced greater recall and F1 scores than XGBoost across all three horizons. This finding further emphasises the strong temporal persistence of the thermal-storage state and demonstrates that increasing model complexity did not automatically improve operational warning performance.

Importantly, these results represent retrospective classification of future temperature states using historical observations only. No fuel-feed, Air1, Air2, or other boiler control setting was modified, and no counterfactual temperature trajectory following a hypothetical intervention was estimated. Consequently, the present analysis supports conclusions regarding forecasting and warning capability, but not regarding causal improvements in boiler stability, fuel consumption, or control performance.

## Conclusions

This study investigated the temporal response characteristics and multi-horizon predictability of the P1 thermal-storage-tank temperature using long-term field data from a biomass-fired boiler system. Robust lag analysis showed that the relationships between combustion-related operational inputs and P1 temperature were statistically detectable but weak and heterogeneous. The aggregate cross-correlation patterns and daily lag distributions therefore did not support interpretation of the system using a single deterministic thermal response time.

Benchmark forecasting further showed that increasing model complexity did not necessarily improve predictive performance. The Linear AR model achieved the lowest RMSE and the highest R^2^ at all three forecasting horizons, while the persistence model remained highly competitive, particularly in terms of MAE. XGBoost provided comparable performance at the 60-min horizon but did not outperform the simpler temporal models at 120 or 180 min. These results indicate that the current and recent historical P1 states account for a substantial proportion of the system’s forecastability, a finding that was also supported by the SHAP analysis of the XGBoost models.

Forecasting performance deteriorated progressively with increasing horizon, whereas coverage of the observed daily lag distributions increased. The 60-min horizon provided the strongest pointwise predictive performance but covered a smaller fraction of the observed delayed responses. The 120-min horizon increased lag coverage while retaining moderate predictive capability, whereas the 180-min horizon provided only a modest additional increase in lag coverage accompanied by a further loss of predictive accuracy. Consequently, no single horizon can be regarded as universally optimal; the appropriate horizon depends on the relative importance assigned to forecast accuracy, warning lead time, and coverage of delayed system responses.

The retrospective out-of-band analysis further demonstrated that forecasting accuracy and warning performance should be evaluated separately. For XGBoost, recall decreased from 0.5343 at 60 min to 0.1761 at 120 min and 0.0538 at 180 min, while specificity increased from 0.9095 to 0.9546 and 0.9854, respectively. Thus, longer-horizon forecasts generated fewer false alarms but detected progressively smaller proportions of actual future out-of-band states. In particular, the 180-min horizon did not provide sufficiently reliable event-detection performance to support strong claims regarding early-warning capability.

Importantly, the present study evaluates predictive and retrospective warning capability rather than the causal effect of control interventions. No fuel-feed or blower setting was modified during the evaluation, and no counterfactual temperature trajectory following a hypothetical intervention was generated. Therefore, the results should not be interpreted as evidence that the proposed forecasting framework directly improves boiler stability, energy consumption, or control performance. Instead, the framework provides quantitative information on future thermal-state risk that may support subsequent development of operator-oriented warning and control strategies.

The findings should also be interpreted within the limitations of the present dataset, which was obtained from a single biomass-fired boiler facility over a limited observation period. Differences in boiler configuration, fuel characteristics, thermal demand, circulation conditions, seasonal operation, and control logic may affect both lag behaviour and forecasting performance. Future work should therefore include external validation at independent facilities, evaluation under different operating regimes and seasons, and prospective real-time testing of explicitly defined intervention rules before the framework is used for closed-loop control or operational optimisation.

## Supporting information

S1 File Public reproducibility package for multi-horizon biomass-boiler forecasting. ZIP archive containing derived non-sensitive datasets, model comparison results, thermal lag analyses, retrospective warning evaluation results, analysis scripts, model configurations, and documentation. The original facility-level operational records are not included because they are subject to third-party data-sharing restrictions.
(ZIP)
